# Higher amyloid correlates to greater loneliness during the COVID-19 pandemic

**DOI:** 10.12688/f1000research.124891.1

**Published:** 2022-10-04

**Authors:** Abigail Kehrer-Dunlap, Rebecca Bollinger, Szu-Wei Chen, Audrey Keleman, Regina Thompson, Anne Fagan, Beau Ances, Susan Stark

**Affiliations:** 1 Program in Occupational Therapy, Washington University in St. Louis School of Medicine, St. Louis, Missouri, 63108, USA; 2 Department of Neurology, Washington University in St. Louis School of Medicine, St. Louis, Missouri, 63108, USA; 3 Knight Alzheimer Disease Research Center, Washington University in St. Louis School of Medicine, St. Louis, Missouri, 63108, USA

**Keywords:** loneliness, Alzheimer disease, preclinical Alzheimer disease, COVID-19

## Abstract

**Background:** Little is known about psychosocial characteristics, including loneliness, anxiety, and depression, present in preclinical Alzheimer disease (AD). The purpose of this cross-sectional study was to examine the relationship between these psychosocial characteristics and amyloid accumulation in cognitively normal older adults with and without preclinical AD during the COVID-19 pandemic.

**Methods:** A global Clinical Dementia Rating ^^®^^ Scale score of 0 was required for enrollment. Cortical amyloid burden was measured using [11C] Pittsburgh compound B or [18F]-Florbetapir PET tracers. Centiloids were used to synchronize measures. Demographic characteristics and measures of loneliness, anxiety, and depression were collected via self-report. Spearman’s correlation was used to examine relationships between amyloid and psychosocial characteristics.

**Results:** The 108 participants had a mean age of 75.0 and an average amyloid burden of 22.2. Higher amyloid accumulation was significantly associated with greater loneliness.

**Conclusions:** Additional research is needed with a larger, more diverse sample to examine these psychosocial characteristics in preclinical AD.

## Introduction

Loneliness is a growing epidemic affecting millions of older adults and is associated with poor physical health, depression, anxiety, mortality, and an increased risk of Alzheimer disease (AD; Malhotra *et al.*, 2021; Sundström *et al.*, 2020; Sutin *et al.*, 2018). Psychosocial characteristics such as loneliness, anxiety, and depression are associated with symptomatic AD ( El Haj *et al.*, 2016; Pietrzak *et al.*, 2015), but it is unclear whether these emerge during the preclinical phase of AD. Individuals with preclinical AD have Alzheimer pathology, including abnormal cerebrospinal fluid (CSF) amyloid-β 1-42 (Aβ _1-42_), total tau (t-tau), and/or phosphorylated tau (p-tau _181_) biomarkers or higher global amyloid burden indices beginning years before the onset of cognitive decline ( Dubois *et al.*, 2016). Individuals with preclinical AD are cognitively normal (CN) and show no clinical symptomology consistent with AD, such as memory loss or hallucinations, for years or even decades ( Price & Morris, 1999); however, other clinical symptoms, such as loneliness, anxiety, and depression, may already be prevalent.

Limited research with CN older adults has shown that greater loneliness is associated with higher amyloid burden ( Donovan *et al.*, 2016) and higher tau pathology ( d’Oleire Uquillas *et al.*, 2018). Research is mixed on the associations between anxiety, higher amyloid burden ( Pietrzak *et al.*, 2015), and higher tau pathology ( d’Oleire Uquillas *et al.*, 2018). The relationship between depression and amyloid-β levels in CN older adults remains unclear; while some studies report that increased amyloid burden measured by positron emission tomography (PET) is linked to depression ( Wu *et al.*, 2014), others report that amyloid PET is not ( Babulal *et al.*, 2020). Further evidence indicates that emotional and psychological distress may accelerate the onset of clinical symptoms of AD for older adults with preclinical AD ( Dubois *et al.*, 2016; Wilson *et al.*, 2007). The ongoing coronavirus disease 2019 (COVID-19) pandemic has led to an increase in the number of older adults reporting loneliness, anxiety, and depression ( Reppas-Rindlisbacher *et al.*, 2021); as a result, older adults with preclinical AD may be at an increased risk for exhibiting clinical symptoms of AD. Additional research is needed to understand how at-risk groups, including individuals with preclinical AD, may be impacted by prolonged emotional and psychological distress throughout the COVID-19 pandemic.

To date, relationships between amyloid burden and loneliness, anxiety, and depression in CN older adults using multiple PET tracers have not been investigated. It is important to understand whether these psychosocial characteristics impact older adults with higher amyloidosis consistent with preclinical AD to identify potential risk factors that may exacerbate the progression of AD. The purpose of this study was to examine psychosocial characteristics (loneliness, anxiety, and depression) in CN older adults during the COVID-19 pandemic using a continuous measure of cortical amyloid burden.

## Methods

### Participants

Community-dwelling older adults were recruited for this cross-sectional study from an ongoing longitudinal cohort study at the Knight Alzheimer Disease Research Center (Knight ADRC) at Washington University in St. Louis ( Bollinger *et al.*, 2021). The inclusion criteria for the cohort study were: (1) ≥65 years old, (2) CN as determined by a Clinical Dementia Rating ^®^ (CDR; Morris, 1993) score of 0, and (3) had CSF biomarkers and/or neuroimaging (PET and/or MRI) within two years of study enrollment. Individuals were excluded if they had a medical or psychiatric diagnosis that could interfere with longitudinal follow-up or neuroimaging or adversely affect cognition. Participants included in this secondary analysis had amyloid PET data processed within two years of study enrollment. The Knight ADRC recruitment procedures have been published previously ( Morris *et al.*, 2019).

### Recruitment

At the beginning of the COVID-19 pandemic, individuals were approached by phone or e-mail and invited to participate. Written informed consent was obtained, and participants completed surveys between 27 April 2020 and 8 June 2020 regarding their mental health with staff via phone or secure e-mail using a Research Electronic Data Capture (REDCap; Harris *et al.*, 2009) survey link. Upon completion, participants could elect to enter a drawing for a $50 gift card. This study was approved by the Institutional Review Board at Washington University in St. Louis (reference number: 201807135).

### Measures

The global CDR score ( Morris, 1993) from the participant’s most recent annual clinical visit at the Knight ADRC was used to assess the level of impairment from normal to severe symptomatic AD. The CDR is scored as follows: 0 (CN), 0.5 (very mild dementia), 1 (mild dementia), 2 (moderate dementia), and 3 (severe dementia). A CDR score of 0 was required for enrollment in this study.

PET amyloid imaging was used to obtain AD biomarker data through previously described methods ( Su *et al.*, 2013, 2015). Participants were injected with either [11C] Pittsburgh compound B (PiB) or [18F]-Florbetapir (AV45), and dynamic scans were acquired. The post-injection time window for quantification was 30–60 minutes for PiB and 50–70 minutes for AV45. A PET Unified Pipeline was implemented to process the data. In short, a region of interest (ROI) segmentation approach was used with FreeSurfer 5.3 (Martinos Center for Biomedical Imaging, Charlestown, MA, USA). Based on the ROI segmentation, a tissue mask was created, and partial volume correction was used ( Su *et al.*, 2013). For each ROI, a standard uptake ratio (SUVR) was acquired with the cerebellar gray matter serving as a reference region. Cortical regions affected by AD were used to obtain a summary value (mean cortical uptake ratio) of the partial-volume corrected SUVR ( Mintun *et al.*, 2006). Centiloids were used to synchronize measures from both PiB and AV45 tracers, with global amyloid deposition scaled from 0–100. Centiloid cutoffs for PiB and AV45 mean cortical SUVR were 16.4 and 20.6, respectively ( Klunk *et al.*, 2015). A value higher than these cutoffs is considered preclinical AD.

Participants answered questions related to the COVID-19 pandemic to capture temporally relevant personal and social characteristics. Demographic characteristics and six COVID-19-related questions were collected via self-report. Annual income was recoded to reflect the federal poverty level ( Office of the Assistant Secretary for Plannning and Evaluation, 2020) and median household income for adults over 65 in the U.S. ( Semega *et al.*, 2020): <$20,000 (low income), $20,000–$50,000 (below U.S. median), and over $50,000 (above U.S. median). COVID-19-related questions included whether the participant had been diagnosed with COVID-19, had practiced social distancing, and four questions to assess financial strain related to the pandemic: “Since the start of the COVID-19 pandemic in the U.S., have you (1) involuntarily lost a job; (2) taken a cut in wage, salary, self-employed income, or similar; or (3) newly applied for public assistance or unemployment?” Participants also rated their financial stress on a scale from 0 (no financial stress) to 10 (severe financial stress).

Loneliness was measured using version 1 of the 20-item UCLA Loneliness Scale ( Russell, 1996). Participants reported how often they experienced subjective feelings of isolation, feeling left out, and lacking companionship on a scale from 1–5. Total scores on this measure range from 20–80, with lower scores indicating less loneliness. This instrument has high internal consistency (0.96) and a test-retest correlation of 0.73. In our sample, internal consistency was high, with Cronbach’s α = 0.95.

Anxiety was measured using the Patient-Reported Outcomes Measurement Information System (PROMIS) Emotional Distress–Anxiety short form 4a ( Pilkonis *et al.*, 2011) and the Hospital Anxiety and Depression–Anxiety subscale (HADS-A; ( Zigmond and Snaith, 1983). The PROMIS Emotional Distress–Anxiety short form 4a measures feelings of being fearful, overwhelmed, and uneasy over the past seven days on a scale from 1 (never) to 5 (always). Total raw scores are converted to uncorrected t-scores and range from 40.3–81.6, with a mean score of 50 and standard deviation of 10. Higher scores indicate greater anxiety. This measure has demonstrated content validity across diverse populations ( Pilkonis *et al.*, 2011). The HADS-A measures how often participants have felt tense, frightened, and restless over the past week on a scale from 1 (not at all or only occasionally) to 5 (most of the time or very often; ( Zigmond and Snaith, 1983). Total scores range from 0–21, with scores greater than 10 indicating clinically meaningful anxiety. This measure has excellent reliability (intraclass correlation coefficient = 0.92) and good internal consistency (Cronbach’s α = 0.87; ( Djukanovic *et al.*, 2017). In our sample, internal consistency measures for the PROMIS and HADS-A were Cronbach’s α = 0.79 and 0.76, respectively.

Feelings of depression were assessed using the Patient Health Questionnaire 9 (PHQ-9; ( Kroenke *et al.*, 2001). The PHQ-9 includes all nine *Diagnostic and Statistical Manual Fourth Edition* ( American Psychiatric Association, 2000) diagnostic criteria for major depressive disorder and measures how often a participant has felt bothered by symptoms such as little interest in doing things, trouble concentrating, and feeling bad about oneself in the past two weeks on a scale from 0 (not at all) to 3 (nearly every day). Scores on the PHQ-9 range from 0–27, with higher scores indicating more severe depression ( Kroenke *et al.*, 2001). The PHQ-9 has internal reliability (Cronbach’s α = 0.86–0.89), excellent test-retest reliability, and established criterion and construct validity ( Kroenke *et al.*, 2001). Internal reliability in our sample was Cronbach’s α = 0.75.

### Statistical analyses

Data were analyzed using SPSS 27.0 (IBM Corp., Armonk, NY; IBM Corp., 2017). Descriptive analyses were performed to examine demographics, COVID-19-related questions, and psychosocial characteristics (loneliness, anxiety, and depression). As amyloid accumulation distribution was skewed, nonparametric Spearman correlations were used to examine the association between a continuous measure of amyloid accumulation and psychosocial characteristics of loneliness, anxiety, and depression.

## Results

Of the 169 participants enrolled in a longitudinal cohort study at Knight ADRC, 150 enrolled in this study, and 108 with amyloid PET data available were included in the analysis. Participants were 75.0 ± 5.5 years old, 53.7% female, and 89.8% non-Hispanic White; 71.3% lived with others, 60.2% were married or partnered, and participants had completed an average of 16.7 years of education. Only one individual in the study reported contracting COVID-19; over 96% of participants reported practicing social distancing to minimize potential exposure to COVID-19. The majority of participants reported annual incomes higher than the U.S. median and had not involuntarily lost a job, sustained a cut in income, newly applied for public assistance, or experienced a high level of financial stress. Mean amyloid accumulation centiloid was 22.2 ± 31.9 (median = 9.2). Table 1 provides a summary of demographic characteristics and COVID-19-related questions.

**Table 1.** Demographic and clinical characteristics of participants.

Variable	Parameter	Value
Age (years)	Mean (SD), range	75.0 (5.5), 66–92
Centiloid	Mean (SD), range Median	22.2 (31.9), -8.2–154.4 9.2
Gender, female	N (%)	58 (53.7)
Race		
Non-Hispanic White	N (%)	97 (89.8)
African American	N (%)	10 (9.3)
Two or more races	N (%)	1 (0.9)
Annual income range		
<$20,000	N (%)	8 (7.4)
$20,000–$50,000	N (%)	32 (29.6)
>$50,000	N (%)	67 (62)
Living situation		
Lives alone	N (%)	31 (28.7)
Lives with others	N (%)	77 (71.3)
Marital status		
Married/partnered	N (%)	65 (60.2)
Not married/partnered	N (%)	43 (39.8)
Education, years	Mean (SD), range	16.7 (2.1), 11–20
COVID-19 positive	N (%)	1 (0.9)
Practiced social distancing	N (%)	104 (96.3)
Involuntarily lost a job	N (%)	2 (1.9)
Cut in income	N (%)	19 (17.6)
Newly applied for public assistance	N (%)	3 (2.8)
Amount of financial stress ^a^	Mean (SD), range	1.5 (1.9), 0-10
UCLA Loneliness ^b^	Mean (SD), range	31.6 (10.8), 20-63
PROMIS Anxiety ^c^	Mean (SD), range	45.8 (6.9), 40.7-63.3
HADS-A ^d^	Mean (SD), range	2.7 (2.7), 0-12
PHQ-9 ^e^	Mean (SD), range	2.6 (3), 0-15

^a^ Amount of financial stress range 0–10 (higher scores indicate greater financial stress).

^b^ UCLA Loneliness Scale range 20–80 (higher scores indicate greater loneliness).

^c^ Patient-Reported Outcomes Measurement Information System Emotional Distress–Anxiety short form 4a range 40.3–81.6 (higher scores indicate greater anxiety).

^d^ Hospital Anxiety and Depression Scale–Anxiety range 0–21 (scores >10 indicate clinically meaningful anxiety).

^e^ Patient Health Questionnaire 9 range 0–27 (higher scores indicate more severe depression).

**Figure 1.**
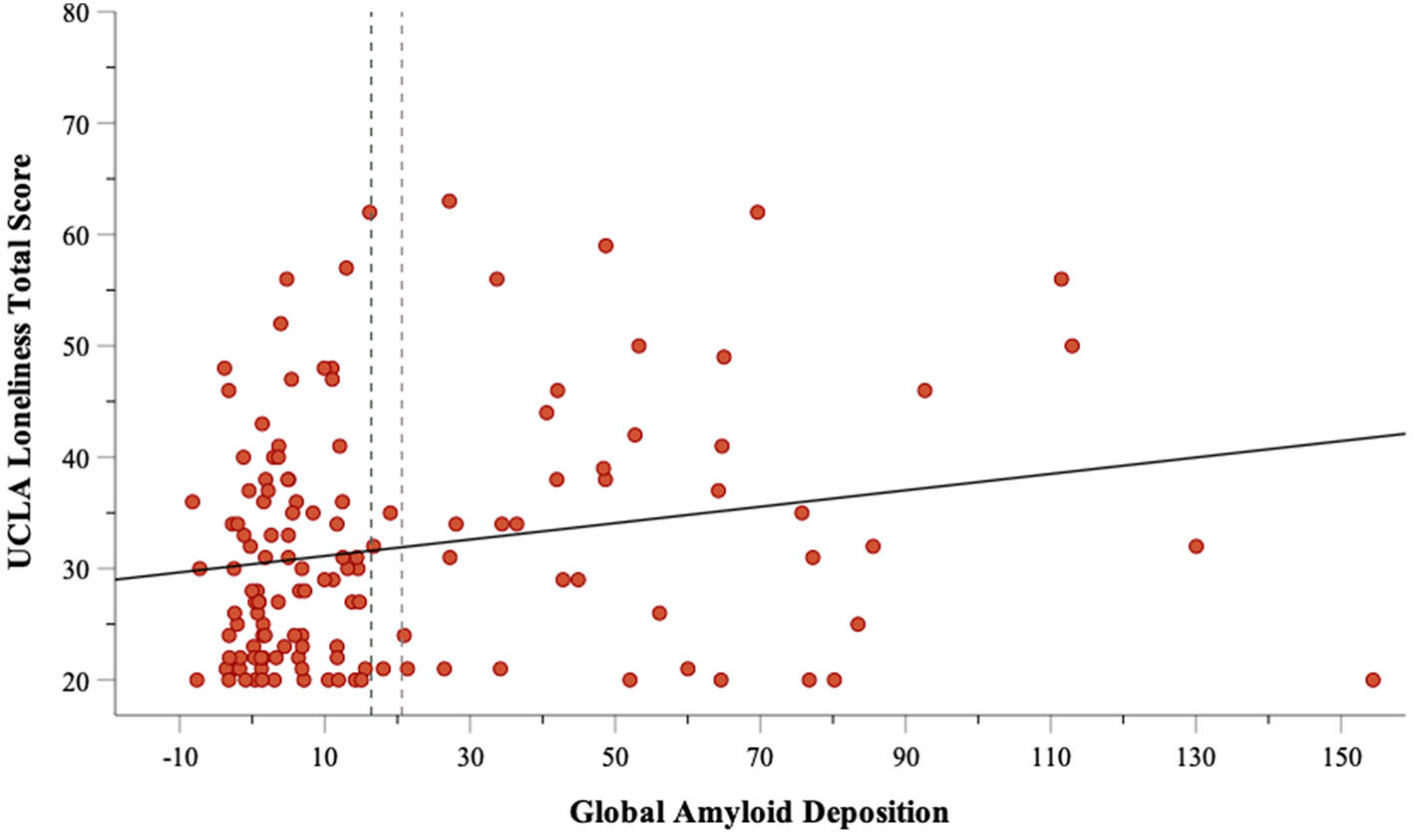
Relationship between global amyloid deposition and UCLA Loneliness total score.

Table 2 displays results of the correlation analysis. Amyloid accumulation was not associated with anxiety or depression. Higher amyloid accumulation was significantly correlated with greater loneliness (rho = .226, *P* = .019); however, this association was considered weak. This relationship is depicted in Figure 1.

**Table 2.** Associations between amyloid accumulation and psychosocial characteristics.

	Centiloid	UCLA Loneliness	PROMIS Anxiety	HADS-A	PHQ-9
Centiloid	—	—	—	—	—
UCLA Loneliness	r = 0.226 * *p* = 0.019 [.03, .4]	—	—	—	—
PROMIS Anxiety	r = 0.123 *p* = 0.205 [-.062, .312]	r = 0.425 *** *p* = 0.000 [.27, .555]	—	—	—
HADS-A	r = 0.190 *p* = 0.05 [.015, .349]	r = 0.502 *** *p* = 0.000 [.334, .645]	r = 0.561 *** *P* = 0.000 [.418, .695]	—	—
PHQ-9	r = 0.088 *p* = 0.367 [-.108, .28]	r = 0.523 *** *p* = 0.000 [.376, .639]	r = 0.478 *** *p* = 0.000 [.323, .618]	r = 0.481 *** *p* = 0.000 [.32, .628]	—

* Indicates *p* < 0.05.

*** Indicates *p* < 0.001.

## Discussion

This study is one of the first to examine loneliness, anxiety, and depression in CN older adults with and without preclinical AD during the early period of the COVID-19 pandemic. We found an association between higher amyloid accumulation and greater loneliness. Although this association was weak, it suggests that older adults with preclinical AD may experience greater loneliness than older adults without preclinical AD. This finding is supported by previous studies showing that loneliness is associated with Alzheimer pathology ( d’Oleire Uquillas *et al.*, 2018; Donovan *et al.*, 2016) and may contribute to AD-related cognitive decline ( Wilson *et al.*, 2007) among CN older adults. Anxiety and depression were not significantly associated with amyloid burden; it is possible that the short timeframe assessed by these questionnaires (past week, past two weeks) did not capture feelings of anxiety and depression that may have occurred at the beginning of the COVID-19 pandemic, prior to the survey. In contrast, the UCLA Loneliness Scale does not include a specific timeframe and may have captured feelings experienced over the several months prior to survey.

Knowledge of these emotional and psychological symptoms is crucial as the clinical symptomology for preclinical AD, aside from neural pathology, is poorly understood. Furthermore, the physical and psychosocial effects of the COVID-19 pandemic will persist far beyond the conclusion of the pandemic. Healthcare professionals should be aware of these symptoms and anticipate care for older adults. Thus, it is imperative to continue examining psychosocial symptoms and the impact of prolonged loneliness on older adults with preclinical AD.

A strength of this study is that data were collected within a short timeframe during the COVID-19 pandemic. In addition, nearly all of our participants reported practicing social distancing, thus implying that they were operating in similar restricted social behaviors.

One limitation of this study is the lack of long-term follow-up, which could provide information about participants’ feelings of loneliness, anxiety, and depression throughout the pandemic, as well as progression of preclinical AD. Another limitation is the lack of diversity in income, ethnicity, and race in our sample. While the demographic characteristics of our participants mimic those of the Knight ADRC, our recruitment source, future research is needed with a wider distribution of income and more diverse sample to examine the generalizability of our findings.

## Conclusions

Despite these limitations, this study points to the importance of psychosocial characteristics in the preclinical phase of AD. Healthcare professionals should monitor for characteristics of loneliness in older adults and provide treatments as needed to mitigate the effects. Future research should be conducted with a larger, more diverse sample over time to examine the relationship between these psychosocial symptoms, accelerated cognitive decline, and AD progression in older adults.

## Data availability

### Underlying data

The full dataset supporting the findings of this study could not be made publicly available as some of the data was collected by the Knight Alzheimer Disease Research Center (ADRC) who requires a separate request. This can be made through ADRC’s website: https://knightadrc.wustl.edu/data-request-form/. In their request, applicants will need to provide the following information: purpose, background and preliminary data, methods, inclusion and exclusion criteria, analytic plan, sample size justification and a list of data variables. A list of variables available for request from the Knight ADRC may be found on their website: https://knightadrc.wustl.edu/professionals-clinicians/request-center-resources/guidelines-data-available/.

Digital Commons@Becker: Higher amyloid correlates to greater loneliness during the COVID-19 pandemic. https://doi.org/10.48765/m113-0447 ( Kehrer-Dunlap *et al*., 2022).

This project contains the dataset of psychosocial measures.

### Extended data

Digital Commons@Becker: Higher amyloid correlates to greater loneliness during the COVID-19 pandemic. https://doi.org/10.48765/m113-0447 ( Kehrer-Dunlap *et al*., 2022).

This project contains the following extended data:

- Falls&AD_Amyloid_Loneliness_Data_Dictionary-akd.csv (data key)
- COVIDQuestions.pdf (COVID items questionnaire)
- COVIDfinancialimpact.pdf (financial impact questionnaire)
- README.txt

Data are available under the terms of the Creative Commons Attribution 4.0 International license (CC-BY 4.0).
