# Higher amyloid is associated with greater loneliness among cognitively normal older adults during the COVID-19 pandemic

**DOI:** 10.12688/f1000research.124891.2

**Published:** 2024-01-24

**Authors:** Abigail Kehrer-Dunlap, Rebecca Bollinger, Szu-Wei Chen, Audrey Keleman, Regina Thompson, Anne Fagan, Beau Ances, Susan Stark

**Affiliations:** 1 Program in Occupational Therapy, Washington University in St. Louis School of Medicine, St. Louis, Missouri, 63108, USA; 2 Department of Neurology, Washington University in St. Louis School of Medicine, St. Louis, Missouri, 63108, USA; 3 Knight Alzheimer Disease Research Center, Washington University in St. Louis School of Medicine, St. Louis, Missouri, 63108, USA

**Keywords:** loneliness, Alzheimer disease, preclinical Alzheimer disease, COVID-19

## Abstract

**Background:**

Loneliness has been associated with several consequences, including increased risk of developing Alzheimer disease (AD). Loneliness may arise during the preclinical phase of AD, but little is known about the relationship between loneliness and amyloid accumulation consistent with preclinical AD. Therefore, the purpose of this study was to examine the relationship between amyloid accumulation and subjective experiences of loneliness among cognitively normal older adults during the COVID-19 pandemic.

**Methods:**

A global Clinical Dementia Rating ^^®^^ Scale score of 0 was required for enrollment. Cortical amyloid burden was measured using [11C] Pittsburgh compound B or [18F]-Florbetapir PET tracers. Centiloids were used to synchronize measures. Demographic characteristics and measures of loneliness, anxiety, and depression were collected via self-report. Multiple linear regression was used to examine the relationship between loneliness and amyloid accumulation.

**Results:**

The 108 participants had a mean age of 75.0 and an average amyloid accumulation of 22.2 ± 31.9. Mean UCLA Loneliness Scale scores were 31.6 ± 10.8. A significant positive association was detected between loneliness and amyloid accumulation (β = 0.064, SE = 0.027, 95% CI = [0.011, 0.118], p = 0.018).

**Conclusions:**

These findings highlight the relationship between higher amyloid accumulation and greater loneliness during the COVID-19 pandemic. Healthcare professionals should include routine assessments for characteristics of loneliness in routine clinical evaluations and integrate loneliness reduction and prevention treatments among older adults experiencing loneliness. Additional research is needed with a larger, more diverse sample to examine the relationship between loneliness and amyloid accumulation.

## Introduction

Loneliness is a growing epidemic affecting millions of older adults and is associated with poor physical health, depression, anxiety, mortality, and an increased risk of Alzheimer disease (AD; Malhotra *et al.*, 2021; Sundström *et al.*, 2020; Sutin *et al.*, 2018). The impacts of loneliness among older adults with AD have been associated with AD symptoms, including hallucinations and an increased rate of cognitive decline ( El Haj *et al.*, 2016; Pietrzak *et al.*, 2015; Wilson *et al.*, 2007). Emerging research suggests that subjective experiences of loneliness may precede clinical symptoms of AD during the preclinical phase of AD ( d’Oleire Uquillas *et al.*, 2018; Donovan *et al.*, 2016). Individuals with preclinical AD are cognitively normal (CN) and show no hallmark symptoms of AD, such as memory loss or hallucinations, for years or even decades ( Price & Morris, 1999). However, these individuals present with Alzheimer pathology, including abnormal amyloid accumulation, that can be reliably characterized using neuroimaging, cerebrospinal fluid, and blood plasma ( Klunk *et al.*, 2004; Schindler *et al.*, 2018, 2019; Wong *et al.*, 2010).

Evidence indicates that emotional and psychological distress may accelerate the onset of clinical symptoms of AD for older adults with preclinical AD ( Dubois *et al.*, 2016; Wilson *et al.*, 2007). It is important to understand how clinical symptoms, such as loneliness, may manifest during the preclinical phase of AD in order to improve our understanding of disease progression.

The ongoing coronavirus disease 2019 (COVID-19) pandemic has led to prolonged social isolation and an increase in the number of older adults reporting loneliness ( Reppas-Rindlisbacher *et al.*, 2021). We sought to characterize subjective experiences of loneliness among our older adult participants during the early months of the pandemic, particularly among those at risk for progressing from preclinical to symptomatic AD.

To date, the relationship between loneliness and amyloid accumulation in CN older adults during the COVID-19 pandemic has not been investigated. As it is important to understand the role of loneliness as a potential risk factor that may exacerbate the progression of AD, the purpose of this study was to examine the relationship between amyloid accumulation and subjective experiences of loneliness among CN older adults during the COVID-19 pandemic.

## Methods

### Participants

Community-dwelling older adults were recruited for this cross-sectional study from an ongoing longitudinal cohort study at the Knight Alzheimer Disease Research Center (Knight ADRC) at Washington University in St. Louis ( Bollinger *et al.*, 2021). The inclusion criteria for the cohort study were: (1) ≥65 years old, (2) CN as determined by a Clinical Dementia Rating ^®^ (CDR; Morris, 1993) score of 0, and (3) had CSF biomarkers and/or neuroimaging (PET and/or MRI) within two years of study enrollment. Individuals were excluded if they had a medical or psychiatric diagnosis that could interfere with longitudinal follow-up or neuroimaging or adversely affect cognition. Participants included in this secondary analysis had amyloid PET data processed within two years of study enrollment. The Knight ADRC recruitment procedures have been published previously ( Morris *et al.*, 2019).

### Recruitment

At the beginning of the COVID-19 pandemic, individuals were approached by phone or e-mail and invited to participate. Written informed consent was obtained, and participants completed surveys between 27 April 2020 and 8 June 2020 regarding their mental health with staff via phone or secure e-mail using a Research Electronic Data Capture (REDCap; Harris *et al.*, 2009) survey link. Upon completion, participants could elect to enter a drawing for a $50 gift card. This study was approved by the Institutional Review Board at Washington University in St. Louis (reference number: 201807135).

### Measures

The global CDR score ( Morris, 1993) from the participant’s most recent annual clinical visit at the Knight ADRC was used to assess the level of impairment from normal to severe symptomatic AD. The CDR is scored as follows: 0 (CN), 0.5 (very mild dementia), 1 (mild dementia), 2 (moderate dementia), and 3 (severe dementia). A CDR score of 0 was required for enrollment in this study.

PET amyloid imaging was used to obtain AD biomarker data through previously described methods ( Su *et al.*, 2013, 2015). Participants were injected with either [11C] Pittsburgh compound B (PiB) or [18F]-Florbetapir (AV45), and dynamic scans were acquired. The post-injection time window for quantification was 30–60 minutes for PiB and 50–70 minutes for AV45. A PET Unified Pipeline was implemented to process the data. In short, a region of interest (ROI) segmentation approach was used with FreeSurfer 5.3 (Martinos Center for Biomedical Imaging, Charlestown, MA, USA). Based on the ROI segmentation, a tissue mask was created, and partial volume correction was used ( Su *et al.*, 2013). For each ROI, a standard uptake ratio (SUVR) was acquired with the cerebellar gray matter serving as a reference region. Cortical regions affected by AD were used to obtain a summary value (mean cortical uptake ratio) of the partial-volume corrected SUVR ( Mintun *et al.*, 2006). Centiloids were used to synchronize measures from both PiB and AV45 tracers, with global amyloid deposition scaled from 0–100. Centiloid cutoffs for PiB and AV45 mean cortical SUVR were 16.4 and 20.6, respectively ( Klunk *et al.*, 2015). A value higher than these cutoffs is considered preclinical AD.

Participants answered questions related to the COVID-19 pandemic to capture temporally relevant personal and social characteristics. Demographic characteristics and six COVID-19-related questions were collected via self-report. Annual income was recoded to reflect the federal poverty level ( Office of the Assistant Secretary for Planning and Evaluation, 2020) and median household income for adults over 65 in the U.S. ( Semega *et al.*, 2020): <$20,000 (low income), $20,000–$50,000 (below U.S. median), and over $50,000 (above U.S. median). COVID-19-related questions included whether the participant had been diagnosed with COVID-19, had practiced social distancing, and four questions to assess financial strain related to the pandemic: “Since the start of the COVID-19 pandemic in the U.S., have you (1) involuntarily lost a job; (2) taken a cut in wage, salary, self-employed income, or similar; or (3) newly applied for public assistance or unemployment?” Participants also rated their financial stress on a scale from 0 (no financial stress) to 10 (severe financial stress).

Loneliness was defined as negative thoughts and feelings of being isolated and disconnected from others ( Russell, Peplau, & Ferguson, 1978). Loneliness was measured using version 1 of the 20-item UCLA Loneliness Scale ( Russell, 1996). Participants reported how often they experienced subjective feelings of isolation, feeling left out, and lacking companionship on a scale from 1–5. Total scores on this measure range from 20–80, with lower scores indicating less loneliness. This measure does not have established cutoff scores to indicate levels of loneliness, and it is recommended to examine the full range of scores when using this measure. This instrument has high internal consistency (0.96) and a test-retest correlation of 0.73. In our sample, internal consistency was high, with Cronbach’s α = 0.95.

Anxiety was defined as feeling nervous, worried, or frightened ( Marks & Lader, 1973). Anxiety was measured using the Patient-Reported Outcomes Measurement Information System (PROMIS) Emotional Distress–Anxiety short form 4a ( Pilkonis *et al.*, 2011) and the Hospital Anxiety and Depression–Anxiety subscale (HADS-A; Zigmond & Snaith, 1983). The PROMIS Emotional Distress–Anxiety short form 4a measures feelings of being fearful, overwhelmed, and uneasy over the past seven days on a scale from 1 (never) to 5 (always). Total raw scores are converted to uncorrected t-scores and range from 40.3–81.6, with a mean score of 50 and standard deviation of 10. Higher scores indicate greater anxiety. This measure has demonstrated content validity across diverse populations ( Pilkonis *et al.*, 2011). The HADS-A measures how often participants have felt tense, frightened, and restless over the past week on a scale from 1 (not at all or only occasionally) to 5 (most of the time or very often; ( Zigmond and Snaith, 1983). Total scores range from 0–21, with scores greater than 10 indicating clinically meaningful anxiety. This measure has excellent reliability (intraclass correlation coefficient = 0.92) and good internal consistency (Cronbach’s α = 0.87; ( Djukanovic *et al.*, 2017). In our sample, internal consistency measures for the PROMIS and HADS-A were Cronbach’s α = 0.79 and 0.76, respectively.

Depression was defined as feelings of sadness, hopelessness, or a lack of interest or pleasure in completing daily activities ( Herrman *et al.*, 2022). Feelings of depression were assessed using the Patient Health Questionnaire 9 (PHQ-9; ( Kroenke *et al.*, 2001). The PHQ-9 includes all nine *Diagnostic and Statistical Manual Fourth Edition* ( American Psychiatric Association, 2000) diagnostic criteria for major depressive disorder and measures how often a participant has felt bothered by symptoms such as little interest in doing things, trouble concentrating, and feeling bad about oneself in the past two weeks on a scale from 0 (not at all) to 3 (nearly every day). Scores on the PHQ-9 range from 0–27, with higher scores indicating more severe depression ( Kroenke *et al.*, 2001). The PHQ-9 has internal reliability (Cronbach’s α = 0.86–0.89), excellent test-retest reliability, and established criterion and construct validity ( Kroenke *et al.*, 2001). Internal reliability in our sample was Cronbach’s α = 0.75.

### Statistical analyses

Data were analyzed using SPSS 27.0 (IBM Corp., Armonk, NY; IBM Corp., 2017). Descriptive analyses were performed to examine demographics, COVID-19-related questions, and psychosocial characteristics (loneliness, anxiety, and depression). Multiple linear regression was used to estimate the associations between loneliness and amyloid accumulation. We controlled for relevant covariates identified through domain knowledge and clinical interest, including anxiety, depression, age, gender, level of financial stress, living situation (alone versus with others), and practice of social distancing, which were added to the model through forced entry. Because the cohort study included two measures of anxiety (PROMIS and HADS-A), we included only the PROMIS Anxiety measure for anxiety in this model given its higher precision in scoring compared to the HADS-A. Ninety-five percent confidence intervals (CIs) for regression coefficients were calculated for all independent variables.

## Results

Of the 169 participants enrolled in a longitudinal cohort study at Knight ADRC, 150 enrolled in this study, and 108 with amyloid PET data available were included in the analysis. Participants were 75.0 ± 5.5 years old, 53.7% female, and 89.8% non-Hispanic White; 71.3% lived with others, 60.2% were married or partnered, and participants had completed an average of 16.7 years of education. Only one individual in the study reported contracting COVID-19; over 96% of participants reported practicing social distancing to minimize potential exposure to COVID-19. The majority of participants reported annual incomes higher than the U.S. median and had not involuntarily lost a job, sustained a cut in income, newly applied for public assistance, or experienced a high level of financial stress. Mean amyloid accumulation was 22.2 ± 31.9 (median = 9.2), and 33 participants (30.6%) were determined to have preclinical AD based on centiloid cutoffs for PiB and AV45; average amyloid accumulation was 4.9 ± 6.3 for participants without preclinical AD and 61.4 ± 32.1 for participants with preclinical AD. Average scores on the UCLA Loneliness Scale were 31.6 ± 10.8, and 46 (42.6%) of participants had total scores that exceeded the sample average. Table 1 provides a summary of demographic characteristics and COVID-19-related questions.

**Table 1.** Demographic and clinical characteristics of participants.

Variable	Parameter	Value
Age (years)	Mean (SD), range	75.0 (5.5), 66–92
Centiloid	Mean (SD), range Median	22.2 (31.9), -8.2–154.4 9.2
Gender, female	N (%)	58 (53.7)
Race		
Non-Hispanic White	N (%)	97 (89.8)
African American	N (%)	10 (9.3)
Two or more races	N (%)	1 (0.9)
Annual income range		
<$20,000	N (%)	8 (7.4)
$20,000–$50,000	N (%)	32 (29.6)
>$50,000	N (%)	67 (62)
Living situation		
Lives alone	N (%)	31 (28.7)
Lives with others	N (%)	77 (71.3)
Marital status		
Married/partnered	N (%)	65 (60.2)
Not married/partnered	N (%)	43 (39.8)
Education, years	Mean (SD), range	16.7 (2.1), 11–20
COVID-19 positive	N (%)	1 (0.9)
Practiced social distancing	N (%)	104 (96.3)
Involuntarily lost a job	N (%)	2 (1.9)
Cut in income	N (%)	19 (17.6)
Newly applied for public assistance	N (%)	3 (2.8)
Amount of financial stress ^a^	Mean (SD), range	1.5 (1.9), 0-10
UCLA Loneliness ^b^	Mean (SD), range	31.6 (10.8), 20-63
PROMIS Anxiety ^c^	Mean (SD), range	45.8 (6.9), 40.7-63.3
HADS-A ^d^	Mean (SD), range	2.7 (2.7), 0-12
PHQ-9 ^e^	Mean (SD), range	2.6 (3), 0-15

^a^ Amount of financial stress range 0–10 (higher scores indicate greater financial stress).

^b^ UCLA Loneliness Scale range 20–80 (higher scores indicate greater loneliness).

^c^ Patient-Reported Outcomes Measurement Information System Emotional Distress–Anxiety short form 4a range 40.3–81.6 (higher scores indicate greater anxiety).

^d^ Hospital Anxiety and Depression Scale–Anxiety range 0–21 (scores >10 indicate clinically meaningful anxiety).

^e^ Patient Health Questionnaire 9 range 0–27 (higher scores indicate more severe depression).

**Figure 1.**
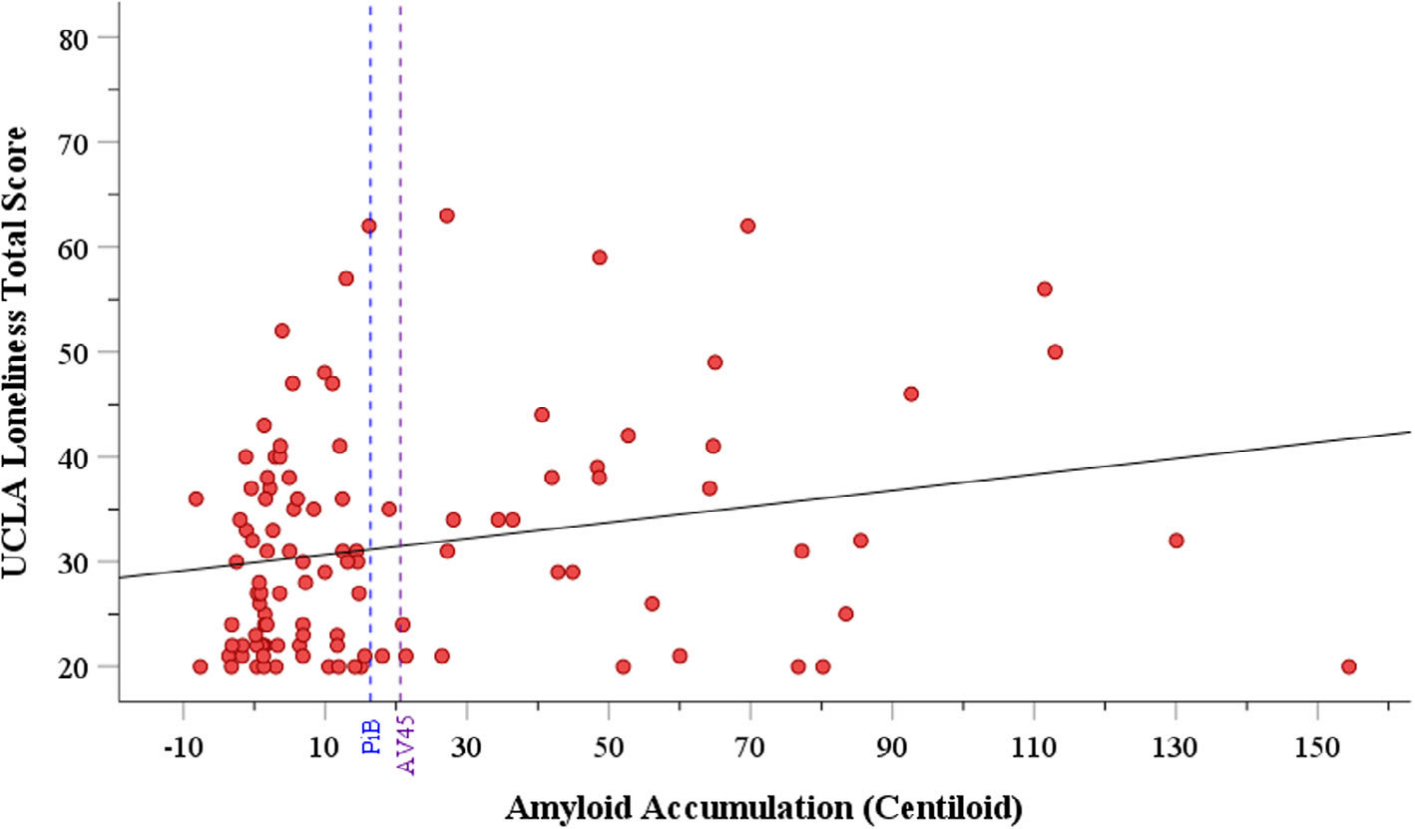
Relationship between amyloid accumulation and UCLA Loneliness total score. Note: Dashed blue line indicates cutoff for preclinical AD positivity using PiB PET tracer (16.4). Dashed purple line indicates cutoff for preclinical AD positivity using AV45 PET tracer (20.6).

Table 2 displays results of the multiple linear regression analysis. The independent variables significantly predicted UCLA Loneliness scores (F(8,99) = 8.69, p < 0.001), and the model explained 41.2% of the variance (R2 = 0.412) in UCLA Loneliness Scale scores. After controlling for anxiety, depression, age, gender, level of financial stress, living situation, and social distancing, we observed a significant positive association between loneliness and amyloid accumulation (β = 0.064, SE = 0.027, 95% CI = [0.011, 0.118], p = 0.018). Figure 1 displays the linear association between loneliness and amyloid accumulation. Significant positive associations were also found between loneliness and depression (β = 1.605, SE = 0.344, 95% CI = [0.923, 2.288], p < 0.001) and level of financial stress (β = 1.157, SE = 0.474, 95% CI = [0.216, 2.097], p = 0.016).

**Table 2.** Multiple linear regression for relationship between UCLA Loneliness score (dependent variable) and amyloid accumulation (centiloid).

	Unstandardized β (SE)	95% CI (lower, upper)	p-value
Centiloid	0.064 (0.027)	0.011, 0.118	0.018
Anxiety			
Age	-0.123 (0.156)	-0.433, 0.187	0.433
Gender	-2.453 (1.790)	-6.005, 1.099	0.174
Living situation	-1.460 (1.959)	-5.346, 2.427	0.458
PROMIS Anxiety	0.236 (0.143)	-0.048, 0.520	0.102
Social distancing	3.011 (4.477)	-5.873, 11.894	0.503
PHQ-9	1.605 (0.344)	0.923, 2.288	<0.001
Financial stress	1.157 (0.474)	0.216, 2.097	0.016

## Discussion

This study is one of the first to examine the relationship between loneliness and amyloid accumulation in CN older adults during the early period of the COVID-19 pandemic. We found an statistically significant association between greater loneliness and higher amyloid accumulation. Although this association was weak, it suggests that older adults with preclinical AD may experience greater loneliness than older adults without preclinical AD. This finding is supported by previous studies showing that loneliness is associated with Alzheimer pathology ( d’Oleire Uquillas *et al.*, 2018; Donovan *et al.*, 2016) and may contribute to AD-related cognitive decline ( Wilson *et al.*, 2007) among CN older adults. A significant positive association was also detected between loneliness and depression; this finding is consistent with previous findings and may be attributed to shared symptomology (e.g., helplessness, unhappiness) between loneliness and depression ( Mushtaq *et al.*, 2014). In addition, there was a significant positive association between loneliness and financial stress; this finding is supported by a previous study indicating a relationship between loneliness and perceived financial concerns as a result of the pandemic ( Bonsaksen *et al.*, 2021).

A strength of this study is that data were collected within a short timeframe during the COVID-19 pandemic. In addition, nearly all of our participants reported practicing social distancing, thus implying that they were operating in similar restricted social behaviors.

However, these findings should be interpreted in light of several limitations. One limitation of this study is the lack of diversity in income, ethnicity, and race in our sample. Our sample was largely not impacted by the pandemic, with the majority reporting low financial stress, having not lost a job, and not needing to apply for public assistance. While the demographic characteristics of our participants mimic those of the Knight ADRC, our recruitment source, future research is needed with a wider distribution of income and more diverse sample to examine the generalizability of our findings. Another limitation is the cross-sectional design lacking long-term follow-up, which could provide information about participants’ subjective experiences of loneliness throughout the pandemic, as well as progression of preclinical AD. In addition, the UCLA Loneliness Scale does not include a specific timeframe and may have captured feelings experienced over the several months prior to survey. Additional research is needed to understand how at-risk groups, including individuals with preclinical AD, may be impacted by prolonged loneliness throughout the COVID-19 pandemic.

Knowledge of these emotional and psychological symptoms is crucial as the clinical symptomology for preclinical AD, aside from neural pathology, is poorly understood. Furthermore, the physical and psychosocial effects of the COVID-19 pandemic will persist far beyond the conclusion of the pandemic. Assessment of characteristics that impact loneliness, including satisfaction with social relationships and opportunities for social participation, should be integrated in routine clinical evaluations of older adults ( National Academies of Sciences, Engineering, and Medicine *et al.*, 2020). Healthcare professionals working with older adults experiencing loneliness should identify appropriate treatments, such as connecting with community resources to offer social support or removing barriers that hinder opportunities for social connectedness ( Gardiner *et al.*, 2018; Perissinotto *et al.*, 2019). They should also adopt prevention approaches to addressing loneliness, including fostering conversations with older adults about maintaining healthy social relationships and including assessments of social activity in annual screenings to monitor social health ( Perissinotto *et al.*, 2019).

## Conclusions

Despite these limitations, this study found an association between loneliness and amyloid accumulation and points to the importance of examining subjective experiences of loneliness during the preclinical phase of AD. Future research should be conducted with a larger, more diverse sample over time to examine the relationship in older adults. Healthcare professionals should monitor for loneliness in older adults and recommend treatments to reduce loneliness and prevent future loneliness.

## Data availability

### Underlying data

The full dataset supporting the findings of this study could not be made publicly available as some of the data was collected by the Knight Alzheimer Disease Research Center (ADRC) who requires a separate request. This can be made through ADRC’s website: https://knightadrc.wustl.edu/data-request-form/. In their request, applicants will need to provide the following information: purpose, background and preliminary data, methods, inclusion and exclusion criteria, analytic plan, sample size justification and a list of data variables. A list of variables available for request from the Knight ADRC may be found on their website: https://knightadrc.wustl.edu/professionals-clinicians/request-center-resources/guidelines-data-available/.

Digital Commons@Becker: Higher amyloid correlates to greater loneliness during the COVID-19 pandemic. https://doi.org/10.48765/m113-0447 ( Kehrer-Dunlap *et al*., 2022).

This project contains the dataset of psychosocial measures.

### Extended data

Digital Commons@Becker: Higher amyloid correlates to greater loneliness during the COVID-19 pandemic. https://doi.org/10.48765/m113-0447 ( Kehrer-Dunlap *et al*., 2022).

This project contains the following extended data:

- Falls&AD_Amyloid_Loneliness_Data_Dictionary-akd.csv (data key)
- COVIDQuestions.pdf (COVID items questionnaire)
- COVIDfinancialimpact.pdf (financial impact questionnaire)
- README.txt

Data are available under the terms of the Creative Commons Attribution 4.0 International license (CC-BY 4.0).
