# Supplementary material for: Higher amyloid is associated with greater loneliness among cognitively normal older adults during the COVID-19 pandemic
Source: F1000Res. 2024 Jan 24;11:1134. Originally published 2022 Oct 4. [Version 2] doi: 10.12688/f1000research.124891.2 (PMC11491702; doi:10.12688/f1000research.124891.2)
Supplement: Peer review history

# Reviewer report: Reviewer response for version 2

**DOI:** 10.5256/f1000research.161373.r240424

**Published:** 2024-02-15

**Authors:** Hui-Fen Mao

**Affiliations:** 1 School of Occupational Therapy, College of Medicine, National Taiwan University, Taipei, Taiwan

This article uses secondary data to demonstrate the relationship between higher amyloid accumulation and greater loneliness among older adults during the COVID-19 pandemic. The results significantly increased credibility after utilizing a multiple linear regression analysis by controlling the relevant covariates.

 The revised article is well-written and addresses the concerns raised in the previous comments. The only suggestion is as follows.

 This study stands out for its focus on loneliness among older adults during the COVID-19 pandemic. However, it remains unclear whether the level of loneliness was more severe during that time compared to other periods. In the revision, the authors reported that a portion of participants (46%) had total scores exceeding the sample average. However, using this percentage may not fully address the issue mentioned above. Despite the absence of established cutoff scores on the UCLA 20-item Loneliness Scale indicating the presence or absence of loneliness, there are two possible methods to clarify this point. First, to report the severity level of loneliness according to the most commonly used categorization: 20–34 denotes a low degree of loneliness, 35–49 a moderate degree of loneliness, 50–64 a moderately high degree, and 65–80 a high degree of loneliness (Deckx *et al.*, 2014 ^1^). Another way is to compare the mean score with previous data from this cohort study rather than data from the time of the COVID-19 pandemic or other studies with similar participants’ backgrounds.

Is the work clearly and accurately presented and does it cite the current literature?

Partly

If applicable, is the statistical analysis and its interpretation appropriate?

Partly

Are all the source data underlying the results available to ensure full reproducibility?

Yes

Is the study design appropriate and is the work technically sound?

Partly

Are the conclusions drawn adequately supported by the results?

Partly

Are sufficient details of methods and analysis provided to allow replication by others?

Yes

Reviewer Expertise:

Occupational Therapy, Long-tern Care, Dementia Care, Assistive technology & Applications

I confirm that I have read this submission and believe that I have an appropriate level of expertise to confirm that it is of an acceptable scientific standard.

## References

[1] : Risk factors for loneliness in patients with cancer: a systematic literature review and meta-analysis. *Eur J Oncol Nurs* .2014;18(5) : 10.1016/j.ejon.2014.05.002 466-77 10.1016/j.ejon.2014.05.002 24993076

# Reviewer report: Reviewer response for version 2

**DOI:** 10.5256/f1000research.161373.r240423

**Published:** 2024-02-15

**Authors:** Paula V Nersesian

**Affiliations:** 1 School of Nursing, University of Southern Maine, Portland, Maine, USA

The analysis of this study has been updated. The authors indicate that for the loneliness measure, “...it is recommended to examine the full range of scores when using this measure.” Although it is not clear who they are suggesting recommends this as no citation is provided. Establishing a cut point for participants who are experiencing a clinically significant manifestation of loneliness and those who are not would provide a clear basis for analysis, but they do not opt for this analysis. Based on the distribution shown in Figure 1, there appears to be very little clinically significant loneliness in this population of participants. The mean is reported as 31.6 +/- 10.8 within a range of 20-80. Even though 42.6% of the participants had values above the mean, it appears from Figure 1 that a majority of responses were close to the mean; very few were experiencing loneliness in a way that might be perceived as clinically significant. These data, taken together, demonstrate little clinically significant loneliness among the study participants. Thus, drawing conclusions about the presence of loneliness and its association with other variables may be permissible based on the values resulting from the linear regression analysis, but the analysis may have very little clinical relevance when the level of loneliness is negligible. Thus, the results stemming from this study must be considered very preliminary and exploratory. Further investigation of these relationships is warranted using a cut point for the loneliness variable.

Is the work clearly and accurately presented and does it cite the current literature?

Partly

If applicable, is the statistical analysis and its interpretation appropriate?

I cannot comment. A qualified statistician is required.

Are all the source data underlying the results available to ensure full reproducibility?

Partly

Is the study design appropriate and is the work technically sound?

Yes

Are the conclusions drawn adequately supported by the results?

Partly

Are sufficient details of methods and analysis provided to allow replication by others?

Yes

Reviewer Expertise:

Loneliness

I confirm that I have read this submission and believe that I have an appropriate level of expertise to confirm that it is of an acceptable scientific standard.

# Reviewer report: Reviewer response for version 1

**DOI:** 10.5256/f1000research.137129.r207323

**Published:** 2023-09-20

**Authors:** Paula V Nersesian

**Affiliations:** 1 School of Nursing, University of Southern Maine, Portland, Maine, USA

This article summarizes a secondary analysis of an investigation of psychosocial factors and their association with biomarkers of preclinical Alzheimer disease. The significant finding was an association of higher amyloid and a measure of loneliness.

 This is a well-written already published, but not yet peer-reviewed article. It lacks basic definitions of the psychosocial factors that it is exploring and in particular, the factor that was shown to have a significant association, loneliness. Although loneliness is the key factor showing a significant association with the amyloid biomarker variable, the authors fail to report the prevalence of loneliness among the study participants. Likewise, the authors fail to discuss the fact that a majority of the study participants (77%) “lives with others” in the discussion section. Is the significant finding therefore being drawn from a minority of study participants? The reader cannot know since the prevalence of loneliness is not reported.

 The conclusion section should become part of an expanded discussion section. It would be helpful if more concrete information were provided to the reader regarding the signs of loneliness that healthcare professionals should monitor for. Suggesting providing “treatments as needed to mitigate effects” is not as helpful as providing concrete suggestions. You may wish to consult this recent publication for ideas as to what to suggest: https://www.hhs.gov/sites/default/files/surgeon-general-social-connection-advisory.pdf

 We do know that it is a biased sample lacking in diversity and the authors duly identify that limitation. The authors report that the data are not publicly available, but special arrangements can be made to access it.

Is the work clearly and accurately presented and does it cite the current literature?

Partly

If applicable, is the statistical analysis and its interpretation appropriate?

I cannot comment. A qualified statistician is required.

Are all the source data underlying the results available to ensure full reproducibility?

Partly

Is the study design appropriate and is the work technically sound?

Yes

Are the conclusions drawn adequately supported by the results?

Partly

Are sufficient details of methods and analysis provided to allow replication by others?

Yes

Reviewer Expertise:

Loneliness

I confirm that I have read this submission and believe that I have an appropriate level of expertise to confirm that it is of an acceptable scientific standard, however I have significant reservations, as outlined above.

# Response

**Published:** 2024-01-16

**Authors:** Susan Stark

**Affiliations:** Washington University in St. Louis, USA

We thank the reviewer for their time and for providing detailed points of feedback. Point-by-point responses to reviewer comments are provided below.

1. This article summarizes a secondary analysis of an investigation of psychosocial factors and their association with biomarkers of preclinical Alzheimer disease. The significant finding was an association of higher amyloid and a measure of loneliness.
  This is a well-written already published, but not yet peer-reviewed article. 
**RESPONSE:** We thank the reviewer for their time and for providing us with several suggestions to increase the clarity of this manuscript.
2. It lacks basic definitions of the psychosocial factors that it is exploring and in particular, the factor that was shown to have a significant association, loneliness.
**RESPONSE:** We have added operational definitions of loneliness, anxiety, and depression in each of their respective methods paragraphs:
  “Loneliness was defined as negative thoughts and feelings of being isolated and disconnected from others (Russell, Peplau, & Ferguson, 1978).”
  “Anxiety was defined as feeling nervous, worried, or frightened (Marks & Lader, 1973).”
  “Depression was defined as feelings of sadness, hopelessness, or a lack of interest or pleasure in completing daily activities (Herrman et al., 2022).”
3. Although loneliness is the key factor showing a significant association with the amyloid biomarker variable, the authors fail to report the prevalence of loneliness among the study participants.
**RESPONSE:** Established cutoffs for what is considered “lonely” are not available for the 20-item UCLA Loneliness Scale (Russell, Peplau, & Ferguson, 1978). Therefore, we are not able to report the prevalence of “lonely” versus “not lonely.” Possible scores on this measure range from 20-80, with higher scores indicating greater loneliness. We have added the following language to address this comment:
  “Total scores on this measure range from 20–80, with lower scores indicating less loneliness. This measure does not have established cutoff scores to indicate levels of loneliness, and it is recommended to examine the full range of scores when using this measure.”
  In addition, we have reported the prevalence of UCLA Loneliness Scale scores that exceed the average in our sample:
  “Average scores on the UCLA Loneliness Scale were 31.6 ± 10.8, and 46 (42.6%) of participants had total scores that exceeded the sample average.”
  Reference: Russell, D., Peplau, L. A., & Ferguson, M. L. (1978). Developing a measure of loneliness. *Journal of Personality Assessment*, *42*(3), 290-294.
4. Likewise, the authors fail to discuss the fact that a majority of the study participants (77%) “lives with others” in the discussion section. Is the significant finding therefore being drawn from a minority of study participants? The reader cannot know since the prevalence of loneliness is not reported.
**RESPONSE: **Based on our updated analysis, we examined the association between loneliness and amyloid accumulation using multiple linear regression while controlling for relevant covariates, including living situation (i.e., living with others versus alone) as well as practice of social distancing (social isolation). As reported above (see comment #3), there are not established cutoff scores indicating presence or absence of loneliness for the UCLA 20-Item Loneliness Scale. However, after controlling for other covariates in our multiple linear regression, a significant association was detected between loneliness and amyloid accumulation, which warrants further investigation in future studies. 
5. The conclusion section should become part of an expanded discussion section. It would be helpful if more concrete information were provided to the reader regarding the signs of loneliness that healthcare professionals should monitor for. Suggesting providing “treatments as needed to mitigate effects” is not as helpful as providing concrete suggestions. You may wish to consult this recent publication for ideas as to what to suggest: https://www.hhs.gov/sites/default/files/surgeon-general-social-connection-advisory.pdf
**RESPONSE: **We have added the following in the discussion section to provide more concrete recommendations for addressing concerns with loneliness among older adults:
  “Assessment of characteristics that impact loneliness, including satisfaction with social relationships and opportunities for social participation, should be integrated in routine clinical evaluations of older adults (National Academy of Sciences, Engineering, and Medicine, 2020). Healthcare professionals working with older adults experiencing loneliness should identify appropriate treatments, such as connecting with community resources to offer social support or removing barriers that hinder opportunities for social connectedness (Gardiner et al., 2018; Perissinotto et al., 2019). They should also adopt prevention approaches to addressing loneliness, including fostering conversations with older adults about maintaining healthy social relationships and including assessments of social activity in annual screenings to monitor social health (Perissinotto et al., 2019).”
  In addition, we have amended the conclusion section to reflect the above added language:
  “Despite these limitations, this study found an association between loneliness and amyloid accumulation and points to the importance of examining subjective experiences of loneliness during the preclinical phase of AD. Future research should be conducted with a larger, more diverse sample over time to examine the relationship between loneliness and amyloid accumulation in older adults. Healthcare professionals should monitor for loneliness in older adults and recommend treatments to reduce loneliness and prevent future loneliness.”
6. We do know that it is a biased sample lacking in diversity and the authors duly identify that limitation. The authors report that the data are not publicly available, but special arrangements can be made to access it.

# Reviewer report: Reviewer response for version 1

**DOI:** 10.5256/f1000research.137129.r155218

**Published:** 2022-12-05

**Authors:** Hui-Fen Mao

**Affiliations:** 1 School of Occupational Therapy, College of Medicine, National Taiwan University, Taipei, Taiwan

Thank you for the opportunity to review this manuscript, which reports the relationship between psychosocial characteristics and amyloid accumulation in cognitively normal older adults with and without preclinical AD during the early period of the COVID-19 pandemic. This topic raises the issue of how the emotional condition of the elderly might accelerate the risk of AD, indicated by a higher amyloid burden.

 This is a preliminary study with a cross-sectional design without longitudinal follow-up to examine how psychosocial characteristics affect AD occurrences or cognitive deterioration. The statistical analysis used only univariate analysis without considering confounding factors, nor other possible moderating factors of these psychosocial characteristics and amyloid accumulation (i.e., gender, age, living alone or not, premorbid cerebrovascular disease risk). Although a weak correlation between amyloid accumulation and loneliness was found, the results should be interpreted with caution and should be considered to have limited impact on the overall body of research.

 As the authors mentioned, the lack of diversity in income, ethnicity, and race in the study sample was one limitation: the individuals included, who tended to have higher social and economic status, might not be highly impacted by the COVID-19 pandemic (i.e., in terms of the amount of financial stress) and thus might bias the results.

 Revisions are needed to make findings and implications clearer to readers.

 Specific Points:

Topic: The authors may consider adding the characteristic of the participants (cognitively normal older adults) in the title.

Introduction:

1. Due to the intention and the data collection during the initial phase of the COVID-19 pandemic, the loneliness, anxiety, and depression assessed in this study more accurately resemble the “mood state” while facing social isolation rather than a “trait” (personal characteristic). Therefore, I would suggest using “mood status” instead of “psychosocial characteristics”.  
2. 1 ^st^ Paragraph, second sentence: “Psychosocial characteristics such as loneliness, anxiety, and depression are associated with symptomatic AD (El Haj et al., 2016; Pietrzak et al., 2015), but it is unclear whether these emerge during the preclinical phase of AD.” What does “unclear” mean, here? Please specify, as increasing studies indicate that clinical symptoms, such as loneliness, anxiety, and depression, may already be prevalent in preclinical AD (Lavretsky et al., 2009; Pietrzak et al., 2014).
3. Why were the authors interested in collecting data during the COVID-19 pandemic? Please add some explanations or evidence that the pandemic might aggravate mood problems, specifically for the elderly.
4. The following sentence is unclear: “The purpose of this study was to examine psychosocial characteristics (loneliness, anxiety, and depression) in CN older adults using a continuous measure of cortical amyloid burden during the COVID-19 pandemic.” The authors may rephrase to clarify that the purpose of this study was to examine the *relationship* between psychosocial characteristics and amyloid accumulation in cognitively normal older adults.

Methods

1. The authors collected COVID-19-related questions, information about annual income, and financial stress. How were these data used to analyze and interpret the results?
2. In order to better understand the correlation between mood and amyloid accumulation, it would be more effective to use multiple regression analysis to control the other possible confounding factors (i.e., age, economic condition, financial stress, living alone or not).

Results

1. How many participants were considered to have preclinical AD according to the Centiloid cutoffs for PiB and AV45 mean cortical SUVR? The authors may add the number of participants and their average amyloid accumulation with and without preclinical AD in Table 1 or describe it in the results section.
2. Figure 1. Please annotate the two vertical dotted lines.
3. Are the terms “amyloid accumulation”(the caption of Table 1) and “global amyloid deposition” ( the caption of Figure 1) the same? If so, please make the terminology consistent.

Discussions

1.  The authors may point out that this study’s cross-sectional design lacks consideration of the confounding factors or moderating factors of these psychosocial characteristics and amyloid accumulation; the findings should be explained more conservatively.     
2. Table 2 shows a moderately significant correlation between depression, loneliness, and anxiety. Additional discussions regarding the relationship between them could be included by comparing the results with the relevant literature. For example, loneliness has been considered one significant variable in people with depression (Erzen & Çikrikci, 2018).

Is the work clearly and accurately presented and does it cite the current literature?

Partly

If applicable, is the statistical analysis and its interpretation appropriate?

Partly

Are all the source data underlying the results available to ensure full reproducibility?

Yes

Is the study design appropriate and is the work technically sound?

Partly

Are the conclusions drawn adequately supported by the results?

Partly

Are sufficient details of methods and analysis provided to allow replication by others?

Yes

Reviewer Expertise:

Occupational Therapy, Long-tern Care, Dementia Care, Assistive technology & Applications

I confirm that I have read this submission and believe that I have an appropriate level of expertise to confirm that it is of an acceptable scientific standard, however I have significant reservations, as outlined above.

## References

[1] : The effect of loneliness on depression: A meta-analysis. *Int J Soc Psychiatry* .2018;64(5) : 10.1177/0020764018776349 427-435 10.1177/0020764018776349 29792097

[2] : Depression and anxiety symptoms are associated with cerebral FDDNP-PET binding in middle-aged and older nondemented adults. *Am J Geriatr Psychiatry* .2009;17(6) : 10.1097/jgp.0b013e3181953b82 493-502 10.1097/jgp.0b013e3181953b82 19472439 PMC2709773

[3] : Amyloid-β, anxiety, and cognitive decline in preclinical Alzheimer disease: a multicenter, prospective cohort study. *JAMA Psychiatry* .2015;72(3) : 10.1001/jamapsychiatry.2014.2476 284-91 10.1001/jamapsychiatry.2014.2476 25629787

# Response

**Published:** 2024-01-16

**Authors:** Susan Stark

**Affiliations:** Washington University in St. Louis, USA

We thank the reviewer for their time and for providing detailed points of feedback. Item-by-item responses to each comment are provided below.

Introduction:

1. Due to the intention and the data collection during the initial phase of the COVID-19 pandemic, the loneliness, anxiety, and depression assessed in this study more accurately resemble the “mood state” while facing social isolation rather than a “trait” (personal characteristic). Therefore, I would suggest using “mood status” instead of “psychosocial characteristics”.
**RESPONSE: **We have re-examined the literature to identify the most appropriate terminology for describing the loneliness, anxiety, and depression experienced by our sample. Upon examining the term “mood states,” we determined that this term can include anxiety and depression but traditionally excludes loneliness (McNair, 1992). To address this feedback, we have removed all mention of “psychosocial characteristics” from the manuscript and instead refer to each term (i.e., loneliness, anxiety, and depression).
  Reference: McNair, D. M. (1992). Profile of mood states. Educational and Industrial Testing Service
2. 1 ^st^ Paragraph, second sentence: “Psychosocial characteristics such as loneliness, anxiety, and depression are associated with symptomatic AD (El Haj et al., 2016; Pietrzak et al., 2015), but it is unclear whether these emerge during the preclinical phase of AD.” What does “unclear” mean, here? Please specify, as increasing studies indicate that clinical symptoms, such as loneliness, anxiety, and depression, may already be prevalent in preclinical AD (Lavretsky et al., 2009; Pietrzak et al., 2014).
**RESPONSE:** We have made the following changes to the introduction to provide greater clarity in the context of previous evidence. In addition, we have included changes to the introduction to align with additional requested changes from both reviewers, including a more focused description of the context of loneliness within CN older adults:
  “The impacts of loneliness among older adults with AD have been associated with AD symptoms, including hallucinations and an increased rate of cognitive decline (El Haj et al., 2016; Wilson et al., 2007). Emerging research suggests that subjective experiences of loneliness may precede clinical symptoms of AD during the preclinical phase of AD (Donovan et al., 2016; d’Oleire Uquillas et al., 2018).”
  “Individuals with preclinical AD are cognitively normal (CN) and show no hallmark symptoms of AD, such as memory loss or hallucinations, for years or even decades (Price & Morris, 1991). However, these individuals present with Alzheimer pathology, including abnormal amyloid accumulation, that can be reliably characterized using neuroimaging, cerebrospinal fluid, and blood plasma (Klunk et al., 2004; Schindler et al., 2019; Schindler et al., 2018; Wong et al., 2010). Evidence indicates that emotional and psychological distress may accelerate the onset of clinical symptoms of AD for older adults with preclinical AD (Dubois *et al.*, 2016; Wilson *et al.*, 2007). It is important to understand how clinical symptoms, such as loneliness, may manifest during the preclinical phase of AD in order to improve our understanding of disease progression.”
  “The ongoing coronavirus disease 2019 (COVID-19) pandemic has led to prolonged social isolation and an increase in the number of older adults reporting loneliness (Reppas-Rindlisbacher *et al.*, 2021). We sought to characterize subjective experiences of loneliness among our older adult participants during the early months of the pandemic, particularly among those at risk for progressing from preclinical to symptomatic AD. To date, the relationship between loneliness and amyloid accumulation in CN older adults during the COVID-19 pandemic has not been investigated. As it is important to understand the role of loneliness as a potential risk factor that may exacerbate the progression of AD, the purpose of this study was to examine the relationship between amyloid accumulation and subjective experiences of loneliness among CN older adults during the COVID-19 pandemic.”
3. Why were the authors interested in collecting data during the COVID-19 pandemic? Please add some explanations or evidence that the pandemic might aggravate mood problems, specifically for the elderly.
**RESPONSE: **The following has been added to the introduction to address this point:
  “The ongoing coronavirus disease 2019 (COVID-19) pandemic has led to prolonged social isolation and an increase in the number of older adults reporting loneliness (Reppas-Rindlisbacher *et al.*, 2021). We sought to characterize subjective experiences of loneliness among our older adult participants during the early months of the pandemic, particularly among those at risk for progressing from preclinical to symptomatic AD. To date, the relationship between loneliness and amyloid accumulation in CN older adults during the COVID-19 pandemic has not been investigated. As it is important to understand the role of loneliness as a potential risk factor that may exacerbate the progression of AD, the purpose of this study was to examine the relationship between amyloid accumulation and subjective experiences of loneliness among CN older adults during the COVID-19 pandemic.”
4. The following sentence is unclear: “The purpose of this study was to examine psychosocial characteristics (loneliness, anxiety, and depression) in CN older adults using a continuous measure of cortical amyloid burden during the COVID-19 pandemic.” The authors may rephrase to clarify that the purpose of this study was to examine the relationship between psychosocial characteristics and amyloid accumulation in cognitively normal older adults.
**RESPONSE:** We have rephrased this statement accordingly in addition to tying it in to the previous suggestion:
  “As it is important to understand the role of loneliness as a potential risk factor that may exacerbate the progression of AD, the purpose of this study was to examine the relationship between amyloid accumulation and subjective experiences of loneliness among CN older adults during the COVID-19 pandemic.”

Methods:

1. The authors collected COVID-19-related questions, information about annual income, and financial stress. How were these data used to analyze and interpret the results?
**RESPONSE: **We initially examined these variables to describe the characteristics of our sample. In response to the below comment (see “Methods,” comment #2), we included the practice of social distancing and level of financial stress as covariates in the multiple linear regression to control for COVID-19-related covariates that may have impacted subjective experiences of loneliness.
2. In order to better understand the correlation between mood and amyloid accumulation, it would be more effective to use multiple regression analysis to control the other possible confounding factors (i.e., age, economic condition, financial stress, living alone or not).
**RESPONSE:** We thank the reviewer for this suggestion and agree that this analysis would be more effective for examining the relationship between loneliness and amyloid accumulation. We have included the results of a multiple linear regression examining the relationship between loneliness and amyloid accumulation in Table 2 in place of the previous Spearman correlation table.
  In addition, we have detailed the methods and findings from this analysis in the text:
  “Multiple linear regression was used to estimate the associations between loneliness and amyloid accumulation. We controlled for relevant covariates identified through domain knowledge and clinical interest, including anxiety, depression, age, gender, level of financial stress, living situation (alone versus with others), and practice of social distancing, which were added to the model through forced entry. Because the cohort study included two measures of anxiety (PROMIS and HADS-A), we included only the PROMIS Anxiety measure for anxiety in this model given its higher precision in scoring compared to the HADS-A. Ninety-five percent confidence intervals (CIs) for regression coefficients were calculated for all independent variables.” 
  “Table 2 displays results of the multiple linear regression analysis. The independent variables significantly predicted UCLA Loneliness scores (F(8,99) = 8.69, p < 0.001), and the model explained 41.2% of the variance ( *R ^2^* = 0.412) in UCLA Loneliness Scale scores. After controlling for anxiety, depression, age, gender, level of financial stress, living situation, and social distancing, we observed a significant positive association between loneliness and amyloid accumulation (β = 0.064, SE = 0.027, 95% CI = [0.011, 0.118], p = 0.018). Figure 1 displays the linear association between loneliness and amyloid accumulation. Significant positive associations were also found between loneliness and depression (β = 1.605, SE = 0.344, 95% CI = [0.923, 2.288], p < 0.001) and level of financial stress (β = 1.157, SE = 0.474, 95% CI = [0.216, 2.097], p = 0.016).”

Results:

1. How many participants were considered to have preclinical AD according to the Centiloid cutoffs for PiB and AV45 mean cortical SUVR? The authors may add the number of participants and their average amyloid accumulation with and without preclinical AD in Table 1 or describe it in the results section.
**RESPONSE: **We have added this information to the body of the text:
  “Mean amyloid accumulation was 22.2 ± 31.9 (median = 9.2), and 33 participants (30.6%) were determined to have preclinical AD based on centiloid cutoffs for PiB and AV45; average amyloid accumulation was 4.9 ± 6.3 for participants without preclinical AD and 61.4 ± 32.1 for participants with preclinical AD.”
2. Figure 1. Please annotate the two vertical dotted lines.
**RESPONSE:** We have annotated these lines using color and text in the figure, as well as added text describing these lines in the legend of Figure 1:
  “Note: Dashed blue line indicates cutoff for preclinical AD positivity using PiB PET tracer (16.4). Dashed purple line indicates cutoff for preclinical AD positivity using AV45 PET tracer (20.6).”
3. Are the terms “amyloid accumulation”(the caption of Table 1) and “global amyloid deposition” ( the caption of Figure 1) the same? If so, please make the terminology consistent.
**RESPONSE:** These terms refer to the same thing; “global amyloid deposition” has been removed, and “amyloid accumulation” is now used consistently throughout the manuscript. 

Discussions:

1. The authors may point out that this study’s cross-sectional design lacks consideration of the confounding factors or moderating factors of these psychosocial characteristics and amyloid accumulation; the findings should be explained more conservatively.
**RESPONSE: **We have added the following to address this comment:
  “Another limitation is the cross-sectional design lacking long-term follow-up, which could provide information about participants’ subjective experiences of loneliness throughout the pandemic, as well as progression of preclinical AD. In addition, the UCLA Loneliness Scale does not include a specific timeframe and may have captured feelings experienced over the several months prior to survey. Additional research is needed to understand how at-risk groups, including individuals with preclinical AD, may be impacted by prolonged loneliness throughout the COVID-19 pandemic.
2. Table 2 shows a moderately significant correlation between depression, loneliness, and anxiety. Additional discussions regarding the relationship between them could be included by comparing the results with the relevant literature. For example, loneliness has been considered one significant variable in people with depression (Erzen & Çikrikci, 2018). 
**RESPONSE: **To address the reviewer’s previous comment regarding a multiple linear regression rather than Spearman correlation, Table 2 now reflects the results of the updated analysis. As we detected a significant association between loneliness and depression in addition to amyloid accumulation, we included discussion of relevant literature to offer a possible explanation for this finding:
  “A significant positive association was also detected between loneliness and depression; this finding is consistent with previous findings and may be attributed to shared symptomology (e.g., helplessness, unhappiness) between loneliness and depression (Mushtaq et al., 2014).”
